# Temporal profiling of rumen and hindgut microbiota revealed enterotypes affecting the microbial interactions and assembly in the gut of dairy cows

**DOI:** 10.1093/ismeco/ycaf130

**Published:** 2025-08-02

**Authors:** Yangyi Hao, Youyoung Choi, Jana Seifert, Wei Wang, Ya Jing Wang, Zhijun Cao, Hongjian Yang, Le Luo Guan, Shengli Li

**Affiliations:** State Key Laboratory of Animal Nutrition and Feeding, Beijing Engineering Technology Research Center of Raw Milk Quality and Safety Control, College of Animal Science and Technology, China Agricultural University, Beijing 100193, China; Department of Agricultural, Food and Nutritional Science, University of Alberta, Edmonton, AB T6G 2P5, Canada; Department of Agricultural, Food and Nutritional Science, University of Alberta, Edmonton, AB T6G 2P5, Canada; Faculty of Land and Food Systems, The University of British Columbia, Vancouver, BC V6T 1Z4, Canada; Department of Functional Microbiology of Livestock, University of Hohenheim, Stuttgart, Baden-Württemberg, 70593, Germany; State Key Laboratory of Animal Nutrition and Feeding, Beijing Engineering Technology Research Center of Raw Milk Quality and Safety Control, College of Animal Science and Technology, China Agricultural University, Beijing 100193, China; State Key Laboratory of Animal Nutrition and Feeding, Beijing Engineering Technology Research Center of Raw Milk Quality and Safety Control, College of Animal Science and Technology, China Agricultural University, Beijing 100193, China; State Key Laboratory of Animal Nutrition and Feeding, Beijing Engineering Technology Research Center of Raw Milk Quality and Safety Control, College of Animal Science and Technology, China Agricultural University, Beijing 100193, China; State Key Laboratory of Animal Nutrition and Feeding, Beijing Engineering Technology Research Center of Raw Milk Quality and Safety Control, College of Animal Science and Technology, China Agricultural University, Beijing 100193, China; Department of Agricultural, Food and Nutritional Science, University of Alberta, Edmonton, AB T6G 2P5, Canada; Faculty of Land and Food Systems, The University of British Columbia, Vancouver, BC V6T 1Z4, Canada; State Key Laboratory of Animal Nutrition and Feeding, Beijing Engineering Technology Research Center of Raw Milk Quality and Safety Control, College of Animal Science and Technology, China Agricultural University, Beijing 100193, China

**Keywords:** rumen microbiota, hindgut microbiota, enterotype, inter-individual variations, milk production

## Abstract

It has been reported that rumen microbiota affects the cattle’s milk-yield productivity, but the gut microbiota’s contribution to the individualized performance and its associated mechanism have not been well defined. In this study, microbiota of 222 rumen and hindgut respective samples collected from 74 cows throughout the prepartum, postpartum, and peak-lactation periods were assessed using 16S rRNA gene amplicon analysis and were evaluated whether they affected inter-individual microbial interactions, assembly, functions, and contributed to host milk production and serum parameters. Prevotella-dominated (R-Prevot, n = 27) and Butyrivibrio-dominated (R-Butyri, n = 47) enterotypes were identified for rumen microbiota, and Prevotellaceae_UCG-003-dominated (H-Prevot, n = 33) and Paeniclostridium-dominated (H-Paenic, n = 41) enterotypes were identified for fecal microbiota. Positive cohesion (cooperative behaviour) was higher, while negative cohesion (competitive behavior) was lower in R-Prevot compared to R-Butyri enterotype throughout the three lactation periods. For H-Prevot enterotype, positive cohesion was higher at prepartum and peak-lactation, but lower at postpartum; and negative cohesion was lower at prepartum and postpartum with no difference detected at peak-lactation. Both deterministic and stochastic processes contributed to the rumen and hindgut microbiota assembly process with the proportion of dispersal limitation process being higher in R-Butyri than in R-Prevot, as well as in H-Prevot than in H-Paenic enterotype at peak-lactation. Additionally, the cows with R-Prevot/H-Prevot enterotypes (n = 15) had higher milk yield and lower serum non-esterified fatty acid concentration than the cows with R-Butyri/H-Paenic enterotypes (n = 29) during lactation. These findings provide evidence that enterotype could affect microbial interactions and assembly processes, as well as the cows’ productivity.

## Introduction

Recent integration of rumen and fecal microbiome studies showed that they are both associated with nitrogen efficiency [1] and milk production [2] in dairy cows. Furthermore, rumen microbes were more influenced by feed intake, whereas fecal microbes were more dependent on feed digestibility [2]. Individual variation in rumen microbiota has been identified in dairy cows [3, 4] and they are reported to be related to differences in milk yield and milk protein yield [5–7]. However, the individualized lower gut microbiota of dairy cows and its influence on cow production is not known, although some recent studies show its important role in affecting cow’s health [8]. Recent studies in human microbiome have proposed the concept of “enterotype”, which can classify living organisms based on the composition of their gut microbial communities [9]. The classification of gut microbiota into different “enterotypes”, based on the microbial taxa that are only detected in certain phenotypes, is effective in differentiating the gut microbiota and relation to the inter-individual host phenotype variations [9, 10]. To date, enterotype classification has played a crucial role in identifying beneficial microbes for both human disease diagnosis and enhancing productivity in animals. For example, human fecal enterotypes were identified with key bacterial taxa like *Prevotella* and *Bifidobacterium* linked to healthy individuals but not to those with diabetes [11, 12]. A *Prevotella*-dominant enterotype in feces was reported to be correlated with higher average daily feed intake in pigs [13] and a recent study reported individualized hindgut enterotypes were associated with host physiologic variances such as blood lymphocytes and triglycerides in transition dairy cows [8]. These suggest that using enterotypes could lead to predictive performance for future precision nutrition and management. However, there is still limited information regarding the microbial enterotypes in the rumen and hindgut of the same cow (whether rumen enterotype can affect the hindgut enterotype) and their potential contribution to individualized milk production. Additionally, how enterotype-specific microbial taxa vary across different lactation stages remains unknown. Answering these questions will provide fundamental knowledge to better understand the systematic roles of gut enterotypes in milk production of dairy cows.

It is known that microbiota composition shift can be affected by the microbial community assembly process [14], which is driven by stochastic and deterministic assembly processes [15]. Additionally, microbial interactions such as inter-microbe collaboration and competition can affect microbial community assembly, which further shapes their resistance and/or resilience to environmental shifts [16]. The microbial assembly processes can directly affect the composition and structure of the gut microbiome and as the results, it can affect the rumen and hindgut fermentation in dairy cows. It is not yet known how the microbial communities’ assembly processes and interactions are associated with different gut enterotypes and inter-individual variations in gut fermentation. Understanding the microbial assembly process will provide insights into how enterotype-driven microbial communities are formed and maintained, ultimately contributing to varied milk yield. Taken together, we hypothesized that the cows with propionate-producing (e.g. *Prevotella*, high rumen fermentation efficiency [17]) taxa-dominated enterotype in rumen and beneficial taxa dominated-enterotype in feces had a better milk production performance. Furthermore, cows with these beneficial enterotypes exhibit different microbial assembly processes compared to other enterotypes and are more likely to maintain consistently high milk yields throughout lactation. Therefore, the objective of this study was to identify the milk production-promoting enterotypes in both rumen and hindgut of cows. Furthermore, understanding the assembly process, and inter-microbe interactions within these enterotypes, as well as if such relationships could impact milk production and host physiological parameters, will provide insights into the strategies for manipulation of ruminant rumen and hindgut microbiota in the future.

## Materials and methods

### Animals management and study

The animal study was approved by the China Agriculture University Laboratory Animal Welfare and Animal Experimental Ethical Faculty (protocol: AW81102202–1-1) and conducted at Hefei Dairy Farm, Anhui, China, from November 2020 to June 2021. Seventy-four multiparous dairy cows with similar parity (2.5 ± 0.73) and body condition score (3.25 ± 0.42) were enrolled from days in milk (DIM) -21 to DIM 50. Cows were fed the same diet under each lactation stage at 7:30, 14:30, and 20:30 with free access to feed and water, while diets were adjusted to meet their nutritional needs at different lactation stages [18]. The information on diet ingredients and chemical composition was presented in Data Set S1.

### Samples and data collection

Milk yield was recorded daily from calving until DIM 50. Milk samples were collected at DIM 21 and 50 at 07:00, 14:00, and 20:00, then pooled in a 4:3:3 ratio for composition analysis. Rumen fluid was collected an hour before the morning feeding using an oral gastric tube and stored at −80°C at DIM −21, 21, and 50. Fecal samples were collected from the rectum at the same time as the rumen fluid and also stored at −80°C. Blood samples were collected from the tail vein before morning feeding at DIM −21, 21, and 50, centrifuged at 3500 × g at 4°C for 15 min to obtain serum, and stored at −20°C for further analysis.

### Milk composition, rumen fluid and feces fermentation, and serum parameters measurement

Milk composition, rumen pH, rumen fluid and feces short-chain fatty acids (SCFA), and ammonia nitrogen (NH_3_-N) concentrations were measured following the methods described by Hao et al., [19]. The serum insulin was evaluated using radioimmunoassay (BFM-96, Multi-tube radioimmunoassay, Zhongcheng Co., Nanjing, China). The serum glucose, triglyceride, alanine transferase (ALT), aspartate transaminase (AST), non-esterified fatty acid (NEFA), β-hydroxybutyrate (BHBA), total antioxidant capacity (T-AOC), and superoxide dismutase (SOD) were assessed using the commercial kits (Kaminuo Biology, Co., Nanjing, China) with a ZECEN CLS880 automated biochemistry analyzer (ZeCen Co., Nanjing, China). Serum leptin was detected with ELISA test kits (Tairui Biology, Co., Beijing, China) following the manufacturer’s guidelines.

### Rumen fluid and feces DNA extraction and amplificon sequencing

Total DNA was extracted from rumen fluid and fecal samples using the PowerSoil DNA Isolation Kit (MoBio Laboratories) with mechanical lysis step via bead-beating following the manufacturer’s guidelines. The DNA quantity, purity, and integrity were determined using a NanoDrop 2000 spectrophotometer (Thermo Fisher Scientific) and 1% agarose gel electrophoresis. Because bacteria are the most abundant among the different domains and play a crucial role in both the rumen [20] and hindgut [21] of ruminants, the primer pairs 338f (5’-ACTCCTACGGGAGGCAGCAG-3′) and 806R (5’-GGACTACHVGGGTWTCTAAT-3′) [22] targeting the bacteria partial 16S rRNA gene were used to generate amplicons for each sample. The procedure for amplicon generation was presented in Supplement Text S1. The amplicons were sequenced using Illumina MiSeq platform (Majorbio Bioinformatics Technology Co. Ltd. (Shanghai, China)) (300-bp paired-end reads).

### Sequence data analysis

The raw sequence data were processed using QIIME2 (Version 2022.2) [23]. Quality control, denoising, removal of chimeric sequences, and generation of amplicon sequencing variants (ASVs) were performed using the QIIME2 plugin DADA2 [24]. Taxonomy classification was performed with the feature classifier command in QIIME2 using ASVs against the SILVA database (Version 138.1). The adequacy of sequencing depth was evaluated by Good’s coverage index. Alpha diversity (Shannon: richness) and beta diversity (Weighted UniFrac distance matrix) were calculated using the scripts implemented in QIIME2 with a depth of 15 307, which was the lowest sequence number in the dataset. To avoid spurious results, only highly abundant taxa (relative abundance >0.01%) detected in at least 50% of the animals were included for downstream analysis.

### Identification of bacterial enterotypes

Rumen and hindgut microbial communities were classified into bacterial enterotypes using Jensen–Shannon distance and partitioning around medoid (PAM) clustering method [25] as described in a previous study [9]. Data sets per cow from three periods were averaged before enterotyping to minimize the DIM effects on individual gut microbiota as reported in previous study [8]. The optimal number of enterotypes was determined using the Calinski–Harabasz index (Fig. S1A). Additionally, Jaccard, Kulczynski, and Bray-Curtis distances based silhouette scores (Fig. S1B) and bootstrapping stability analysis (Fig. S1C) were used to validate enterotype robustness. Between-Class analysis identified the taxa contributing most to the variance between enterotypes.

### Network construction and analysis

We further performed network analysis to estimate the inter-microbe interactions within different ruminal and fecal enterotypes. The bacterial networks were constructed using the random matrix theory (RMT)-based approach that determines the correlation cut-off threshold automatically [26]. This RMT-based network tool, the Molecular Ecological Network Analysis Pipeline (MENAP), is available at the Institute for Environmental Genomics, University of Oklahoma (http://ieg4.rccc.ou.edu/MENA/). The calculations for the network topology, identification of network specialists and generalists [27], network robustness [28–30], as well as the competitive and cooperative interactions among taxa [31] were detailed in Supplement Text S1.

### Assessment of microbial community assembly processes

The infer community assembly mechanisms by phylogenetic bin-based null model analysis (iCAMP) framework were used to assess the rumen and hindgut microbial community assembly processes [32]. iCAMP could differentiate the relative importance of five assembly processes including heterogeneous selection (HeS), homogeneous selection (HoS) for deterministic processes, homogenizing dispersal (HD), dispersal limitation (DL), and drift and others (DR) for stochastic processes at both whole community and bin levels [33]. For the construction of ASV bins, ASV with the highest relative abundance was designated as the centroid taxon of the first bin. All taxa with a distance to the centroid taxon less than the phylogenetic signal threshold of 0.2 were assigned to that bin until the bin size was 24 (the bin contains 24 ASVs) [32]. The next bin was generated from the rest of the taxa in the same way. The calculation of the assembly process was presented in Supplement Text S1.

### Statistical analysis

Rumen and hindgut fermentation parameters, milk production, and serum parameters were analyzed using a completely randomized design with repeated measures via the MIXED procedure (SAS 9.4), in which the fixed effects included enterotypes, DIM, and their interactions, with cows as a random effect. The fermentation parameters between enterotypes were further compared using a preplanned t-test. In addition, the variations in gut fermentation, milk production, and serum parameters among cows with different enterotype combinations were assessed using analysis of variance (ANOVA) test. The correlation between rumen and hindgut enterotypes was assessed with Chi-square test. Microbial Shannon index, and relative abundance of genus were analyzed using a linear mixed model (LMM) [34] with “lme4” (v1.1.31) in R, in which the enterotypes, DIM, and their interactions were fixed effects, with cows as random effects. Statistical significance was determined by Wald type II χ^2^ tests, adjusted by the false discovery rate. Effect sizes, defined as the estimated coefficients of fixed effects, were obtained from LMM. Bacterial taxa were classified as individualized (*P* ≤ tests or common (*P* > .05) based on the significance of the enterotype effect. The microbial β diversity was analyzed by permutational multivariate analysis of variance (PERMANOVA) with a Weighted UniFrac distance matrix. Microbial community robustness and cohesion were analyzed with t-tests based on normality and variance homogeneity. The mediation role of gut fermentation between microbial enterotypes and phenotypes (serum parameter and milk yield) was assessed with Bi-directional mediation analysis using the “mediation” package (v4.5.0). The genus with the highest taxon weight represented each microbial enterotype for mediation analysis. A *P* ≤ .05 was considered significant, and.05 < *P* ≤ .10 indicated a tendency.

## Results

### Rumen and hindgut enterotypes and their differences in diversity

For rumen fluid samples, 1 411 686 raw reads (60 413 ± 3458 per sample, mean ± standard error) were obtained, with,455 316 non-chimeric reads (29 078 ± 1697) remaining after quality control. For fecal samples, 1,141 180 raw reads (54 690 ± 3649) were obtained, with 659 334 non-chimeric reads (29 997 ± 2195) remaining. In total, 33 phyla, 226 families, and 407 genera were identified for rumen microbiota, and 25 phyla, 132 families, and 264 genera were identified for hindgut microbiota.

**Figure 1:**
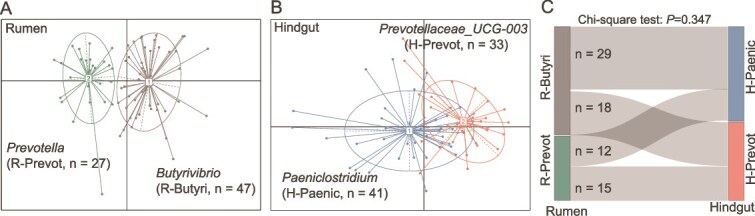
Rumen and hindgut enterotypes of dairy cows within the same herd. The relative abundance of genus was averaged per cow across the three time points resulting in 74 samples included in this analysis. The labels in the figure represent the genus with the highest taxon weight of the enterotype. (A) In rumen enterotype, enterotype1 was dominated by *Butyrivibrio* (R-Butyri, n = 47) and enterotype2 was dominated by *Prevotella* (R-Prevot, n = 27). (B) In hindgut, enterotype1 was dominated by *Paeniclostridium* (H-Paenic, n = 41) and enterotype2 was dominated by *Prevotellaceae_UCG-003* (H-Prevot, n = 33). (C) The number of cows under different combinations of rumen and hindgut enterotypes.

**Figure 2:**
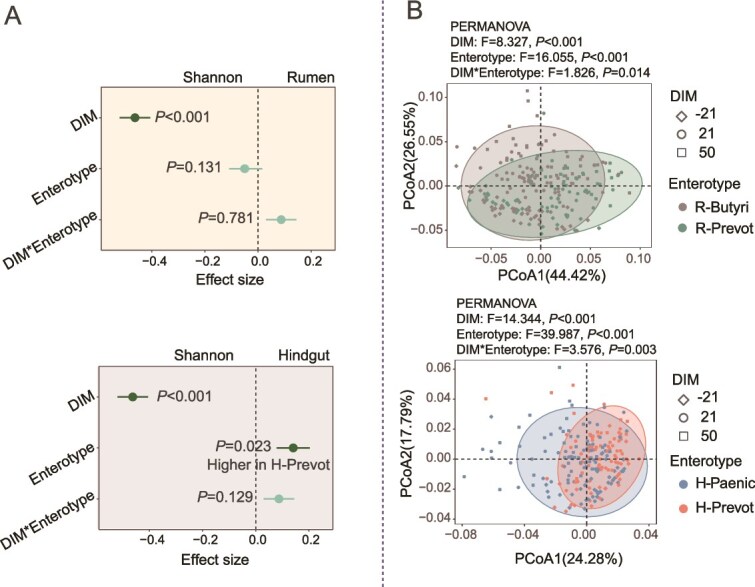
Rumen and hindgut microbiota diversity. (A) Linear mixed model uncovered the effects of enterotypes, DIM, and their interactions on microbiota Shannon index in rumen and hindgut. The enterotypes, DIM, and their interactions worked as fixed effects, cow worked as random effects in the linear mixed model. Statistical significance is based on Wald type II χ^2^ tests. The P values were adjusted by false discovery rate. (B) The microbial β diversity was affected by enterotypes, DIM, and their interactions based on weighted UniFrac distance in rumen and hindgut. DIM: days in milk. PERMANOVA: permutational multivariate analysis of variance.

Among the 74 cows, a total of two rumen enterotypes and two hindgut enterotypes were classified, respectively, and their diversity showed significant differences. The *Prevotella*-dominated (R-Prevot, n = 27) and *Butyrivibrio*-dominated (R-Butyri, n = 47) enterotypes were classified for rumen microbiota (Fig. 1A) and *Prevotellaceae_UCG-003*-dominated (H-Prevot, n = 33) and *Paeniclostridium*-dominated (H-Paenic, n = 41) enterotypes were classified for fecal microbiota (Fig. 1B). Hindgut enterotypes were independent of rumen enterotypes based on the Chi-square test (*P* = .347) (Fig. 1C). Under the different combination of rumen and hindgut enterotypes, 15, 12, 18, and 29 cows were classified into R-Prevot/H-Prevot, R-Prevot/H-Paenic, R-Butyri/H-Prevot, and R-Butyri/H-Paenic enterotypes, respectively. Further comparison of microbial diversity between different rumen or hindgut enterotypes showed that Shannon index was higher (*d* = 0.113, *P* = .023) in H-Prevot enterotype compared to in H-Paenic enterotype (Fig. 2A and Data Set S2). The Shannon index were also different (*d* > 0.40, *P* < .001) with the DIM changed in both rumen and hindgut. However, no significant interaction effect between DIM and enterotypes was observed for Shannon index. Significant disparateness (PERMANOVA *P* < .05) was also observed in different enterotypes, DIM, as well as their interactions with rumen and hindgut microbial communities based on weighted UniFrac distance (Fig. 2B).

### Microbiota composition differed between cows with identified bacterial enterotypes.

**Figure 3:**
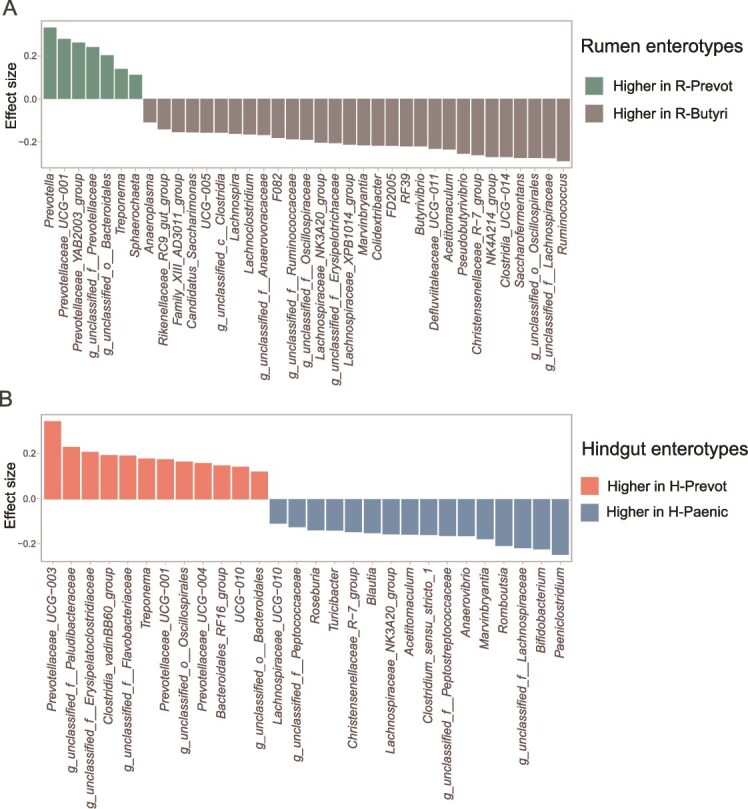
The enterotype specific microbial taxa were identified based on linear mixed model (LMM) in (A) rumen and (B) hindgut, respectively. The enterotypes, DIM, and their interactions worked as fixed effects, cow worked as random effects in the LMM. The taxa were identified as enterotype specific taxa with the fixed effect enterotype *P* < .05. Statistical significance was based on Wald type II χ^2^ tests. The P values were adjusted by false discovery rate. Only the taxa with fixed effect enterotype *P* < .05 were presented in the figure. DIM: days in milk.

In our study, the microbial taxa were assigned into individualized taxa based on mixed model analysis with the enterotype *P* < .05. In the rumen, thirty-seven bacterial genera (69.60%) were classified as individualized taxa (Data Set S3). Among them, seven genera (e.g. *Prevotella*: *d* = 0.332, *P* < .001, *Prevotellaceae_UCG-001*: *d* = 0.279, *P* < .001, *Prevotellaceae_YAB2003_group*: *d* = 0.262, *P* < .001) had higher relative abundances (*P* < .05) in R-Prevot enterotype, while thirty genera (e.g. *Ruminococcus*: *d* = −0.290, *P* < .001, *g_unclassified_f__Lachnospiraceae*: *d* = −0.276, *P* < .001, *g_unclassified_o__Oscillospirales*: *d* = −0.265, *P* < .001) were more abundant in R-Butyri enterotype (Fig. 3A). In hindgut, twenty-eight genera (36.70%) were classified as individualized taxa (Enterotype *P* < .05) (Data Set S3). Among these, 12 genera (e.g. *Prevotellaceae_UCG-003*: *d* = 0.343, *P* < .001, *g_unclassified_f__Paludibacteraceae*: *d* = 0.230, *P* < .001, *g_unclassified_f__Erysipelatoclostridiaceae*: *d* = 0.208, *P* < .001) were more abundant (*P* < .05) in H-Prevot enterotype, while sixteen genera (e.g. *Paeniclostridium*: *d* = −0.244, *P* < .001, *Bifidobacterium*: *d* = −0.220, *P* < .001, *g_unclassified_f__Lachnospiraceae*: *d* = −0.215, *P* < .001) had higher relative abundance in H-Paenic enterotype (Fig. 3B).

### Comparison of microbial interactions in the gut of cows with different enterotypes

**Figure 4:**
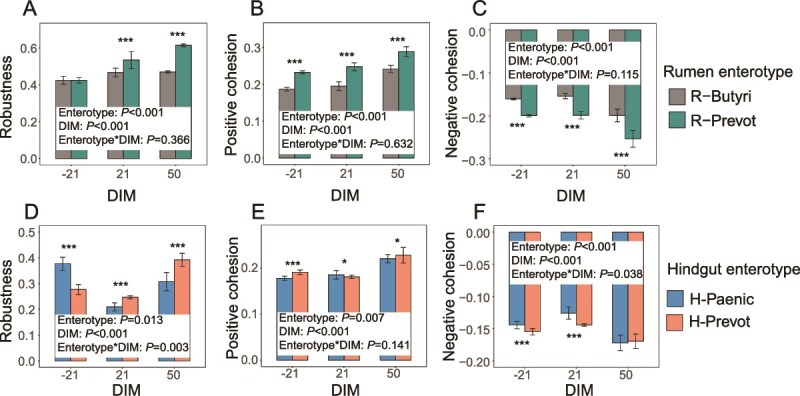
The microbial community robustness and cohesion in different rumen (A–C) and hindgut (D–F) enterotypes. Linear mixed model uncovered the effects of enterotypes, DIM, and their interactions on microbial network’s robustness and cohesion in rumen and hindgut, respectively. The enterotypes, DIM, and their interactions worked as fixed effects, cow worked as random effects in the linear mixed model. Statistical significance is based on Wald type II χ^2^ tests. The P values were adjusted by false discovery rate. T-test was further used to judge the discrepancy of robustness and cohesion among the different enterotypes at different DIM. ^*^: *P* < .05, ^**^: *P* < .01, and ^***^: *P* < .001. DIM: days in milk.

Microbial interactions exhibited distinct patterns in terms of network topology, robustness, and negative/positive cohesion within the rumen and hindgut enterotypes, respectively. In rumen, the node’s average degree, edge number, and edge density were higher in the network of R-Prevot enterotype compared to R-Butyri enterotype throughout the three lactation stages (Data Set S4). There were 16, 11, and 0 generalists observed in the network of R-Prevot enterotype at DIM −21, 21, and 50, respectively. Additionally, 7, 14, and 1 generalists were observed in the network of R-Butyri enterotype at DIM −21, 21, and 50, respectively. The robustness and positive/negative cohesion of microbial networks in the rumen were significantly influenced by both enterotype (*P* < .001) and DIM (*P* < .001), whereas their interaction had no significant effect on these parameters. Specifically, the robustness was higher (*P* < .001) in the network of R-Prevot enterotype compared to R-Butyri enterotype at DIM 21 and 50, while no difference was observed at DIM −21 between the two enterotypes (Fig. 4A). The positive cohesion (Fig. 4B) was higher (*P* < .001), while the negative cohesion (Fig. 4C) was lower (*P* < .001) in R-Prevot compared to R-Butyri enterotype in all three lactation stages.

In the hindgut, the H-Prevot enterotype network had higher edge numbers and average degree than the H-Paenic enterotype across all lactation stages (Data Set S4). The H-Prevot network had 16, 10, and 8 generalists at DIM −21, 21, and 50, respectively, while H-Paenic had 16, 11, and 11 generalists. The robustness and positive/negative cohesion of microbial networks in the hindgut were also significantly influenced by both enterotype (*P* < .001) and DIM (*P* < .001). Moreover, their interaction had a significant effect on robustness (*P* < .001) and negative cohesion (*P* < 0.05). However, no significant effect of the interaction between enterotype and DIM was observed on positive cohesion. Specifically, robustness was higher (*P* < .001) in H-Paenic at DIM −21 but lower (*P* < .001) at DIM 21 and 50 compared to H-Prevot enterotype. Positive cohesion was higher (*P* < .05) in H-Prevot at DIM −21 and 50, but lower (*P* < .05) at DIM 21 than in H-Paenic enterotype (Fig. 4E). In addition, negative cohesion was lower (*P* < .001) in H-Prevot at DIM −21 and 21 than in H-Paenic enterotype, with no difference at DIM 50 (Fig. 4F).

### Assembly processes in different bacterial enterotypes

**Figure 5:**
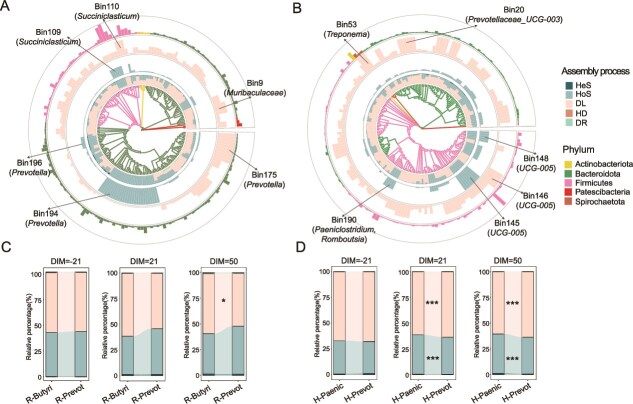
Rumen and hindgut microbial community assembly process. Variation of ecological process across different phylogenetic groups in rumen (A) and hindgut (B). Phylogenetic tree was displayed at the center and only relative abundance >0.1% ASV was presented here. From the inner to outside, the 1st annulus presented the relative abundance of different ecological processes in each bin. The 2rd annulus indicated the contribution of each bin to the whole community’s HOS process. The 3rd annulus presented the contribution of each bin to the whole community’s DL process. The 4th annulus represents the average relative abundance of the ASVs in each bin. For the construction of ASV bins, ASV with the highest relative abundance was designated as the centroid taxon of the first bin. All taxa with a distance to the centroid taxon less than the phylogenetic signal threshold of 0.2 were assigned to that bin until the bin size was 24 (the bin contains 24 ASVs). Microbiota enterotypes assembly process in rumen (C) and hindgut (D). The significant differences between different microbiota enterotypes were calculated by permutational t-test (1000 times). ^*^P < .05, ^**^P < .01, ^***^P < .001. The deterministic process included HeS and HoS. The stochastic process included HD, DL, and DR. DIM: days in milk.

The assembly processes were composed of both the stochastic and deterministic processes in rumen and hindgut microbial enterotypes, respectively. However, it can be different between the different enterotypes. The relative abundance of HoS process (the deterministic process) and DL process (the stochastic process) ranged from 40%–50% and 50%–60% in the two ruminal enterotypes throughout the three lactation stages (Fig. 5C). No difference in assembly process was observed between rumen enterotypes on DIM −21 and 21, while the relative percentage of DL process was higher (*P* < .05) in R-Butyri enterotype than in R-Prevot enterotype at DIM 50. Bin 194 (ASVs belonging to *Prevotella* genus), Bin 109 (ASVs belonging to *Succiniclasticum* genus), and Bin 196 (ASVs belonging to *Prevotella* genus) were the top three bins contributing to HoS process in ruminal bacterial communities (Fig. 5A). Bin 175 (ASVs belonging to *Prevotella* genus), Bin 110 (ASVs belonging to *Succiniclasticum* genus), and Bin 9 (ASVs belonging to *Muribaculaceae* genus) were the top three Bins contributed to DL process in rumen.

In hindgut, the relative abundances of HoS process and DL process were ranged from 30%–40% and 60%–70% in the two enterotypes throughout the three lactation stages (Fig. 5D). A higher HoS (*P* < .001) and lower DL (*P* < .001) process were observed in H-Prevot enterotype compared to H-Paenic enterotype at DIM 21 and 50 (Fig. 5D). The three contributing-most bins to HoS process in hindgut bacterial community were Bin 145 (ASVs belonging to *UCG-005* genus), Bin 190 (ASVs belonging to *Paeniclostridium* and *Romboutsia* genus), and Bin 148 (ASVs belonging to *UCG-005* genus) (Fig. 5B). Bin 146 (ASVs belonging to *UCG-005* genus), Bin 20 (ASVs belonging to *Prevotellaaceae_UCG-003* genus), and Bin 53 (ASVs belonging to *Treponema* genus) were the top three bins contributing to DL process in hindgut.

### Gastrointestinal fermentation parameters between different enterotypes

**Figure 6:**
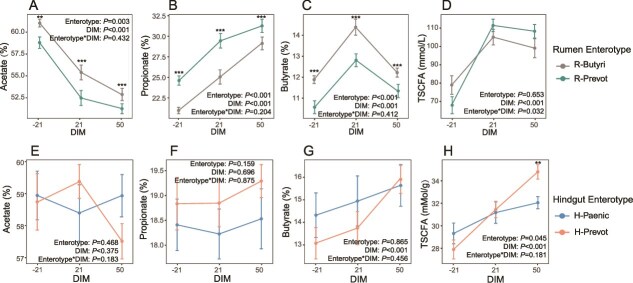
The difference in fermentation parameters between the two enterotypes in rumen (A-D) and hindgut (E-H), respectively. The rumen and hindgut fermentation parameters were analyzed using mixed model. Fixed effects included enterotype, DIM, and their interactions. Cows were included as a random effect. In addition, a preplanned t-test was used to evaluate differences in rumen and hindgut fermentation parameters between different enterotypes at each lactation stage. DIM: days in milk. TSCFA: total short chain fatty acid. ^*^: *P* < .05, ^**^: *P* < .01, and ^***^: *P* < .001.

The fermentation parameters showed differently between the two enterotypes in rumen and hindgut, respectively (Data Set S2). In the rumen, pH, total short-chain fatty acid (TSCFA), NH_3_-N concentrations, and proportions of isobutyrate, isovalerate, and valerate did not differ between the enterotypes (Fig. 6D, Fig. S2A). However, the R-Butyri enterotype had higher (*P* < .01) proportions of acetate, butyrate, and acetate to propionate (A/P) ratio, and a lower propionate proportion than the R-Prevot enterotype across all lactation stages (Fig. 6A-C, Fig. S2A). Comparing rumen fermentation parameters among four groups based on combined rumen and hindgut enterotypes, the R-Prevot/H-Prevot and R-Prevot/H-Paenic groups had lower (*P* < .05) acetate, butyrate, and A/P ratio, and higher propionate proportion than the R-Butyri/H-Prevot and R-Butyri/H-Paenic groups (Fig. S3A).

In the hindgut, TSCFA concentration significantly differed (*P* = .045) between the two enterotypes (Fig. 6H), which was higher in the H-Prevot enterotype than in the H-Paenic enterotype at DIM 50, with no difference at DIM −21 and 21. Proportions of acetate, propionate, butyrate, isobutyrate, valerate, and isovalerate did not differ between hindgut enterotypes or combined rumen and hindgut enterotypes. However, TSCFA concentration was higher (*P* < .05) in R-Prevot/H-Prevot and R-Butyri/H-Prevot cows than in R-Prevot/H-Paenic and R-Butyri/H-Paenic cows at DIM 50 (Fig. S3B).

### Blood, and milk production parameters between different enterotypes

**Figure 7:**
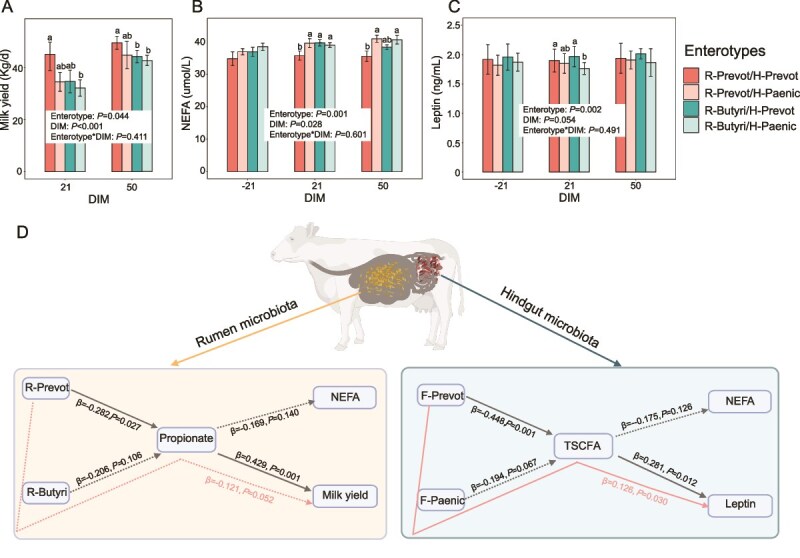
The milk yield (A), serum NEFA (B), and leptin (C) concentrations under different combinations of rumen and hindgut enterotypes in cows. The mediation model disclose the estimated direct and indirect causal relationship between the microbial enterotypes and phenotypes (serum parameters and milk yield) in dairy cows (D). The milk yield, serum NEFA and leptin concentration were analyzed using mixed model. Fixed effects included enterotypes, DIM, and their interactions. Cows were included as a random effect. The analysis of variance (ANOVA) test was employed to assess variations in these parameters among the cows with different combinations of rumen and hindgut enterotypes at each lactation stage. The different letters indicate a significant difference among these groups (*P* < .05). DIM: days in milk. NEFA: non-esterified fatty acid. In the mediation model analysis, genus with the highest taxon weight in each cluster was applied to represent the corresponding enterotype. Solid arrows represented significant (*P* < .05) effects, while dotted line arrows represented non-significant (*P* ≥ .05) effects. Arrows in black color indicated a direct effect, and arrows in pink color indicated a mediation effect that the fermentation parameter mediates the relationship between microbiome enterotypes and phenotypes. The non-significant effects were eliminated unless the pathways were biologically informative.

Cows with the R-Prevot/H-Prevot enterotype combination exhibited better milk production performance and lower levels of blood parameters associated with negative energy balance compared to cows with other rumen and hindgut enterotype combinations. Specifically, no differences were found in milk fat, protein, lactose, and total solids between rumen or hindgut enterotypes (Data Set S5). However, milk yield (*P* = .047) and fat correct milk (FCM) yield (*P* = .039) were higher in R-Prevot than in R-Butyri cows. FCM yield was also higher (*P* = .015) in H-Prevot than in H-Paenic cows. Comparing combinations of enterotypes, milk yield (Fig. 7A) and FCM yield (Fig. S4A) were higher (*P* < .05) in R-Prevot/H-Prevot cows than in R-Butyri/H-Paenic cows at DIM 21 and 50. Additionally, milk yield at DIM 50 and FCM yield at DIM 21 and 50 were lower (*P* < .05) in R-Butyri/H-Prevot cows compared to R-Prevot/H-Prevot cows.

For serum parameters, NEFA concentration was lower (*P* = .013) in R-Prevot cows compared to R-Butyri cows (Data Set S5). Serum BHBA and NEFA concentrations were lower (*P* < .05), and leptin concentration was higher (*P* = .012) in H-Prevot cows compared to H-Paenic cows. In the different combinations of rumen and hindgut enterotypes, NEFA was lower (*P* < .05) in R-Prevot/H-Prevot cows than in other groups at DIM 21 (Fig. 7B). NEFA was also lower (*P* < .05) in R-Prevot/H-Prevot cows compared to R-Prevot/H-Paenic and R-Butyri/H-Paenic cows at DIM 50. BHBA was lower (*P* < .05) in R-Prevot/H-Prevot cows than in R-Butyri/H-Paenic cows at DIM 21 (Fig. S4B). Leptin was higher (*P* < .05) in R-Prevot/H-Prevot and R-Butyri/H-Prevot cows than in R-Butyri/H-Paenic cows at DIM 21 (Fig. 7C). No significant differences were observed in serum glucose, TG, ALT, AST, SOD, T-AOC, or insulin among the different enterotypes throughout the lactation stages (Fig. S4B).

### Associations among enterotypes, fermentation parameters, serum parameters, and milk production

The estimated direct and indirect causal relationship between the microbial enterotypes and phenotypes (serum parameters and milk production) with the fermentation parameter as mediator was further explored (Fig. 7D). R-Prevot enterotype had a direct positive correlation (β = 0.282, *P* = .027) with rumen propionate proportion, which was in turn positively directly associated (β = 0.429, *P* = .001) with milk yield. In hindgut, H-Prevot enterotype had a direct positive correlation (β = 0.448, *P* = .001) with TSCFA concentration, which was in turn positively direct associated with the serum leptin (β = 0.281, *P* = .012). Additionally, H-Prevot enterotype was also indirectly associated (β = 0.126, *P* = .030) with serum leptin concentration with the TSCFA as the mediator.

## Discussion

In this study, we identified rumen and hindgut enterotypes in dairy cows under three lactation stages. Compared to the ruminants’ enterotype-related research, our study assessed the microbial enterotypes of two key digestive regions in cattle, rumen and hindgut, simultaneously. In line with previous studies, the *Prevotella*-dominated enterotype was one of the most common enterotypes in the rumen [35, 36]. However, the *Butyrivibrio*-dominated enterotype has not been reported previously, which was different from reported *Ruminococcus*-dominated enterotype in the previous studies [35, 36]. Rumen microbiota can be affected by many factors such as diet [37], host genetics [38], and lactation stage [6]. The *Ruminococcus*-dominated enterotype was identified in goat kids fed a forage-to-concentrate ratio of 6:4, which contrasts with our study, in which cows were fed a high-grain diet from the prepartum period through peak lactation [35]. Additionally, the *Ruminococcus*-dominated enterotype in dairy cows was identified at 130–150 DIM, corresponding to mid-lactation [36], whereas in our study, cows were sampled from −21 to 50 DIM, covering the transition to peak lactation. This difference in physiological stage suggests distinct gut digesta flow rates and metabolic statuses, which may influence the gastrointestinal microbiota. Moreover, the previous study [36] did not provide detailed dietary information, making it difficult to determine whether the observed difference in enterotype classification was diet-related. However, we speculate that diet could be an important factor contributing to the discrepancy in enterotype distribution. In hindgut, in line with the previous studies [39, 40], bacterial genus that was associated with the *Prevotellaceae* family was one of the hindgut enterotype-related microbial taxa in our study. However, no *Paeniclostridium* dominant enterotype has been reported so far. Interestingly, previous studies reported *Clostridium sensu stricto 1* [39] and unclassified *Clostridiales* [8] dominated enterotypes in the hindgut of ruminants, which belonged to the same bacterial family, Clostridiaceae, as *Paeniclostridium*. These could also be due to the updates in taxa classifications, however, these phylogenetically closely related taxa have similar functional roles [41] and similar environmental preferences [42], suggesting these taxa belonging to the same family can be classified as dominant players in the Clostridiaceae enterotype. It is noticeable that enterotype concept has limitations, which may cause the discrepancy in enterotype classification among different studies. Firstly, the enterotype could be population dependent and/or farm management dependent. Our study did not find the same rumen enterotype reported by Wang et al [35], suggesting a larger population size from multiple farms with similar management is needed to validate the enterotypes found in this study. Secondly, the sampling time could also affect the enterotype assessment as the host physiology status and production stage could be one of another factors that affect the microbial community and the dominant/unique taxa (enterotype) assessment [2]. Lastly, rumen genera *Prevotella* and *Butyrivibrio* have been reported to be significantly correlated with cows’ genotype [43], and it is unclear the relationship between the enterotype-specific taxa and the heritable taxa, especially in fecal microbiota. These suggest that the host genetics could play a role in the enterotypes and future research needs to include the host genetic information (genotypes) to assess the “true” enterotypes differentiating the host-varied phenotypes. Last but not least, while PAM effectively identifies enterotypes based on distance metrics, it may oversimplify microbiome dynamics by forcing discrete clustering. Future studies should integrate multiple clustering methods, such as hierarchical clustering [44], dirichlet multinomial mixtures [45], and/or gradient-based approaches [46], to achieve a more comprehensive view of microbial enterotypes.

In this study, we also found that the variations in microbial diversity and composition in different enterotypes contributed to varied gut fermentation parameters. Together with a higher propionate proportion and a lower A/P ratio in the rumen and higher milk production yield, R-Prevot enterotype could have higher feed efficiency, an extremely important trait affecting dairy cows’ production sustainability. In addition, the identified individualized taxa also played an important role in rumen fermentation. For example, the individualized taxon *Prevotella* is an important fermenter that has a remarkable ability to convert complex carbohydrates to propionate in rumen [17]. The propionate in the rumen can supply substrates for gluconeogenesis in the liver and further support milk synthesis and energy metabolism [47, 48]. The observed higher relative abundance of *Prevotella* in the R-Prevot enterotype may contribute to the higher propionate proportion and lower A/P ratio. Furthermore, the individualized taxon *Butyrivibrio* is an important butyrate producer in the rumen [49], which may contribute to a higher butyrate proportion in the R-Butyri enterotype. The production of butyrate simultaneously leads to hydrogen formation, while propionate constitutes as a hydrogen sink [50], suggesting that different enterotypes may have different hydrogen flow in the rumen. Although we did not measure the methane emission in the current study, the enterotype-driven hydrogen flow suggests the potential contributions of the identified enterotypes in contributing to the variations in methane production. Future studies should investigate the relationship between rumen enterotypes and methane emissions and isotopic tracing of hydrogen utilization pathways. Additionally, metagenomic and metatranscriptomic analyses could provide deeper insights into the functional roles of enterotype-specific microbial taxa in methane metabolism.

The observed higher TSCFA in H-Prevot may be attributed to its higher relative abundance of *Prevotella-UCG-003*, a genus is known for fermenting polysaccharides [20] and producing acetate and propionate [51]. In addition, the higher relative abundance of *Paeniclostridium* in H-Paenic enterotype may suggest the gut dysbiosis which led to lower TSCFA concentration. The lower TSCFA concentration in the hindgut was reported to be associated with diarrhea in transition dairy cows [19]. *Paeniclostridium* is reported to be gut inflammation-related bacterial genus that is negatively associated with the SCFA production [52]. Furthermore, the genus *Paeniclostridium* is an opportunistic pathogenic commensal [52], a higher relative abundance of this taxon may cause the host to have the higher energy expenditure on the immune system [53]. Therefore, assessment of H-Paenic enteryotype could be an effective approach to predict dairy cows’ gut health during early lactation, which could help to prevent severe negative energy balance and health issues in cows.

In this study, we further assessed how the inter-microbes interactions differed between enterotypes. The observed higher edge number, average degree, edge density, and mean distance in the networks of R-Prevot enterotype suggest more interactions among microbial taxa than in the R-Butyri enterotype. Among them, the cooperative and competitive interactions in the microbial community, represented by the positive and negative cohesions, respectively [31] were differed among enterotypes. A higher positive cohesion in R-Prevot enterotype compared to R-Butyri enterotype indicates the microbial community is more cooperative [16], suggesting microbes in R-Prevot enterotype were more robustness and increased the resilience to the abrupt dietary and physiological change during early lactation stage, which helped the cows with a better rumen fermentation function and milk production. In hindgut, a lower negative cohesion in H-Prevot enterotype indicates that the microbial taxa were more competitive than those in the H-Paenic enterotype during the prepartum and postpartum stages. These could be led to less resistance to the dietary shift in the hindgut [16] and this enterotype further promoted the fermentation and SCFA production in the hindgut. All these findings revealed that both rumen and hindgut microbiota can be more adaptive as such have higher milk yield in the R-Prevot/H-Prevot cows.

The observed both HoS and DL processes indicated a combination of deterministic and stochastic assembly processes working synergistically with the microbial assembly in the gastrointestinal tract of dairy cows. DL process represents the restriction for microbial taxa to reach a new niche for colonization, which can constrain them to better grow and utilize nutritional substrates [54]. The lower relative percentage of DL process observed in the R-Prevot enterotype compared to the R-Butyri enterotype during the peak lactation stage suggests that the microbial taxa in the R-Prevot enterotype had more chance to colonize at new niches compared to the R-Butyri enterotype, which may help them to utilize the feedstuff. Lower relative percentage of DL process also promotes microbial interactions [15, 55] and led to more nodes and edges in the networks of R-Prevot enterotype. Lower relative percentage of HoS process in H-Prevot enterotype can lead to less microbial taxa trade-off and/or collaboration [15] and cause more dissimilar structures in microbial communities [56]. Therefore a higher relative percentage of DL process and a lower relative percentage of HoS process indicates there was less chance for microbial taxa to reach a new niche and lead to less microbial trade-off and/or collaboration in the communities of H-Prevot enterotype than in H-Butyri enterotype. The observed ASVs belonging to the generalists (rumen: *Prevotella*, *Succiniclasticum*, *Muribaculaceae*, and hindgut: *Paeniclostridium*) contributed more to the assembly process in terms of their contribution proportion. It is suggested that targeting these microbial taxa could harness the assembly process for a more efficient and productive microbial community in ruminants. More sampling points among the different lactation stages are needed to disclose the assembly process contributing to the different composition and microbial interactions between the distinct enterotypes.

We further identified the microbial interactions, and important individualized taxa that contributed to the distinct fermentation parameters under different enterotypes. Furthermore, the variations in milk yield and serum parameters were influenced by different enterotypes. In line with the previous study, the cows had severe negative energy balance (higher serum NEFA and/or BHBA concentrations), also had a lower serum leptin [57], which was supported by the cows with R-Butyri/H-Paenic enterotype. Additionally, previous studies indicated that the gut microbiota can modulate leptin sensitivity and body fat metabolism in mice [58, 59]. Our study further confirmed that the individualized taxon *Prevotellaceae_UCG-003* in hindgut was indirectly associated with serum leptin via mediating the hindgut SCFA concentration. It has been reported that the gut SCFA can stimulate the leptin production in mice [60]. The observed positive correlation between hindgut TSCFA and serum leptin suggests that microbial-associated SCFA could be also the trigger to regulate serum leptin in dairy cows. However, more animal studies combined with microbiota manipulation are needed to validate these statistically based correlations.

Compared to the previous study reported the rumen [35, 36] and hindgut enterotype [8] separately in dairy cows, we further confirmed that the cows with different combinations of rumen and hindgut enterotypes had distinct milk production. Although there was no significant linkage found between rumen and hindgut enterotypes in our study. Such lack of connection between rumen and hindgut communities can be attributed to the complexity of the gastrointestinal structure and the substantial variability in environmental conditions across different gut regions in ruminants [61]. Rumen and hindgut have different functional roles and they both contribute to host health and metabolism [61]. Therefore, we suggest that the combinations of rumen and hindgut enterotypes could better reflect the crosstalking between gut enterotypes and milk production in dairy cows compared to evaluating rumen and/or hindgut enterotypes separately.

In this study, we used 16S rRNA gene sequencing to characterize microbial profiles; however, several limitations should be acknowledged. First, the 16S rRNA gene lacks the resolution to differentiate closely related microbial species or strains, which may obscure functionally distinct microbes within broad taxa like *Prevotella* or *Butyrivibrio*. Additionally, while this method provides taxonomic classification, it does not directly assess functional genes or metabolic pathways, limiting our ability to link enterotypes to microbial functions such as SCFA production. Primer selection may also introduce biases, as certain taxa could be preferentially amplified while others are underrepresented, potentially skewing enterotype composition. Moreover, 16S rRNA gene sequencing focuses primarily on bacteria and archaea, excluding other important microbial groups such as fungi, protozoa, and viruses, which may play crucial roles in microbial interactions and fermentation. Another limitation is that this approach provides only a static snapshot of microbial communities at a given time point, making it difficult to capture microbial turnover and temporal changes in enterotype composition across different lactation stages. Lastly, because 16S rRNA gene sequencing reports relative abundance rather than absolute microbial counts, functional comparisons between enterotypes, particularly regarding fermentation efficiency and SCFA production, should be interpreted with caution. Finally, even under the same feeding and management system, the feed intake can be different among the cows, which can directly affect the rumen microbiota, as well as the host genes should be taken into consideration for the observed individually varied milk production, a comprehensive study, including feed intake, host genetic information, and metagenome sequencing is supposed to be conducted in the future. Furthermore, a more frequent sampling interval is needed to provide more insights into dynamic microbial shifts throughout the lactation.

## Conclusion

**Figure 8:**
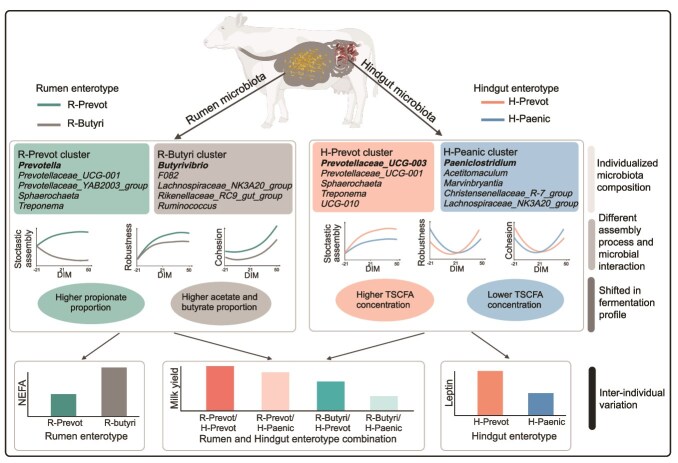
A summary of experimental findings. The different enterotype microbial communities had individualized taxa and different microbial interactions, which further caused the different fermentation parameters in the rumen and hindgut, respectively. All of these contributed to the inter-individual variation in the milk production of dairy cows. TSCFA: total short chain fatty acid. NEFA: non-*esterified* fatty acid. The microbial genus in bold represents the genus with the highest taxon weight of the enterotype.

The present work was the first study that integrated different lactation stages and multi-regions of the gastrointestinal tract to reveal the relevancy between inter-individual variation in microbiota and cows’ production performance (Fig. 8). Our study provided evidence that enterotype could affect microbial interactions and assembly processes, as well as such microbial ecological difference could be linked to inter-individual variation in microbial functions and host phenotypes. Specifically, targeting enterotypes dominated by *Prevotella* or other key taxa through dietary interventions or probiotics could potentially enhance milk production. However, practical application requires further validation, considering the complexity of host-microbiota interactions and environmental factors in dairy farming. Additionally, the findings of this study may be applicable to other ruminants, providing a framework for understanding gut microbial ecology and developing strategies to optimize microbiota for improved productivity across species. However, whether beef cattle have the same enterotype as dairy cattle, needs to be further verified. These findings have enhanced the understanding of gut microbial ecology in dairy cows and the identified individualized rumen and hindgut taxa could be a potential target for mankind to manipulate gut microbiota for a better production performance of cows.

## Supplementary Material

Hao_et_al_Fig_S1_ycaf130

Hao_et_al_Fig_S2_ycaf130

Hao_et_al_Fig_S3_ycaf130

Hao_et_al_Fig_S4_ycaf130

Data_Set_S1_ycaf130

Data_Set_S2_ycaf130

Data_Set_S3_ycaf130

Data_Set_S4_ycaf130

Data_Set_S5_ycaf130

Supplement_Text_S1_ycaf130

Supplement_figures_tables_and_text_information_ycaf130

## Data Availability

The sequencing raw data are deposited in the NCBI Short Read Archive database under accession number PRJNA 1103992.
