# Supplementary figures and images for: Temporal profiling of rumen and hindgut microbiota revealed enterotypes affecting the microbial interactions and assembly in the gut of dairy cows
Source: ISME Commun. 2025 Aug 2;5(1):ycaf130. doi: 10.1093/ismeco/ycaf130 (PMC12376038; doi:10.1093/ismeco/ycaf130)

## A Rumen

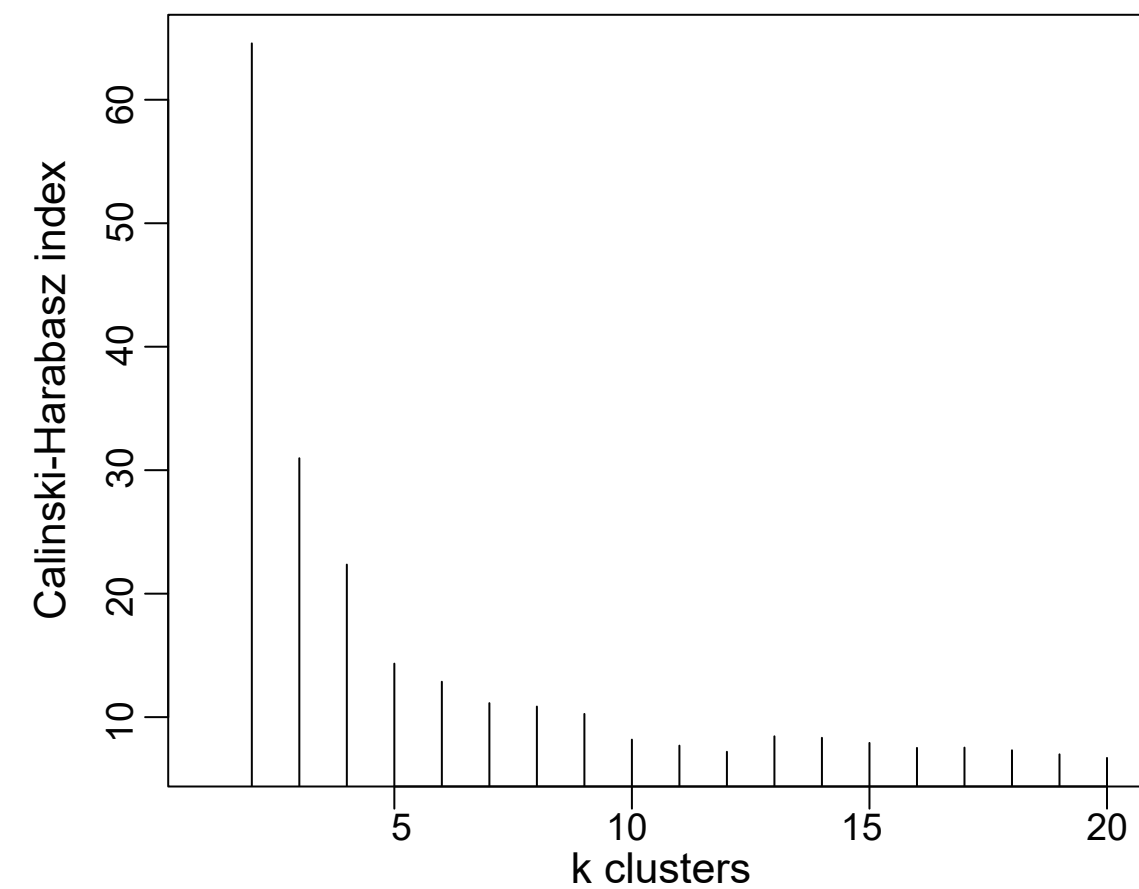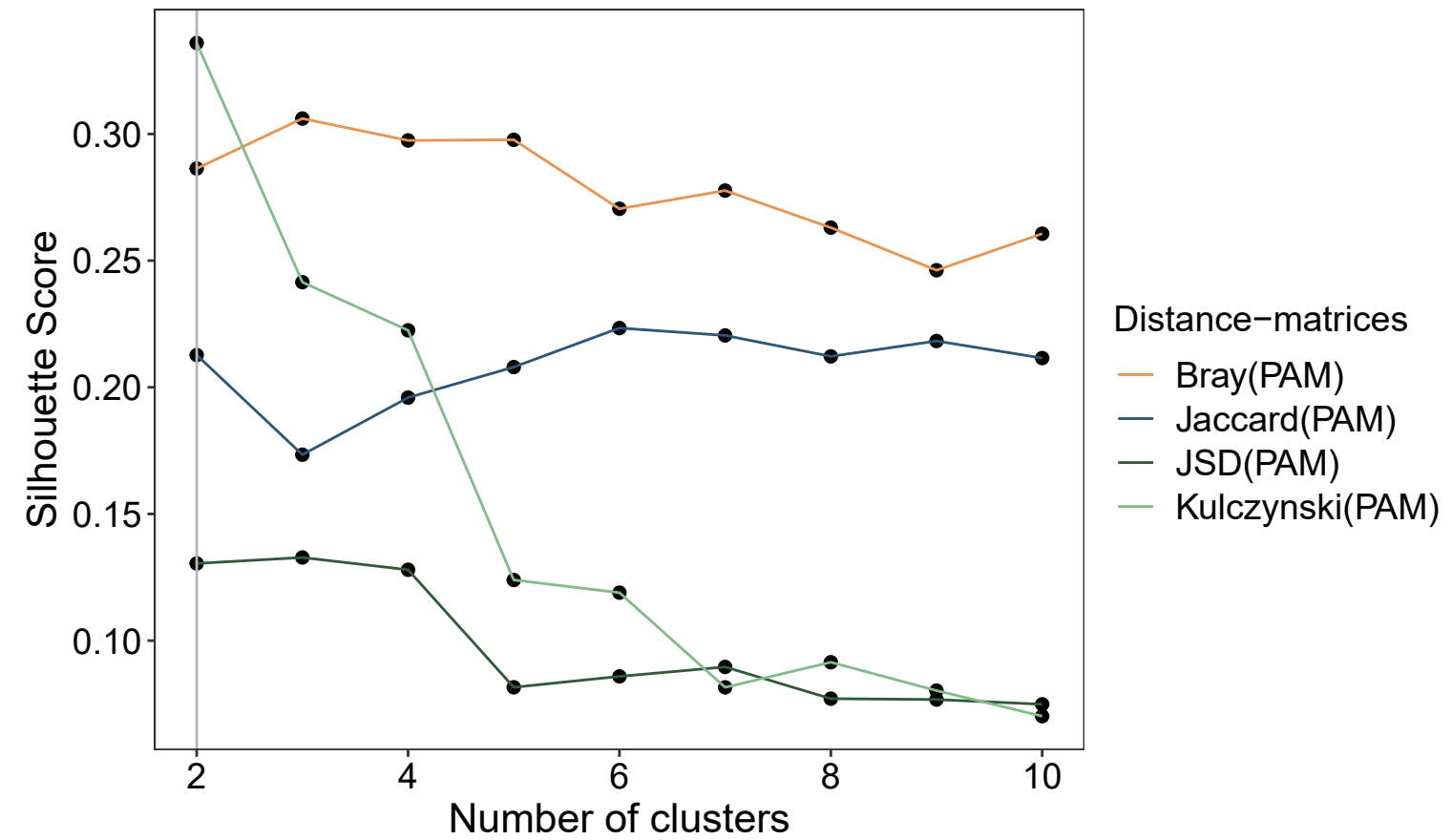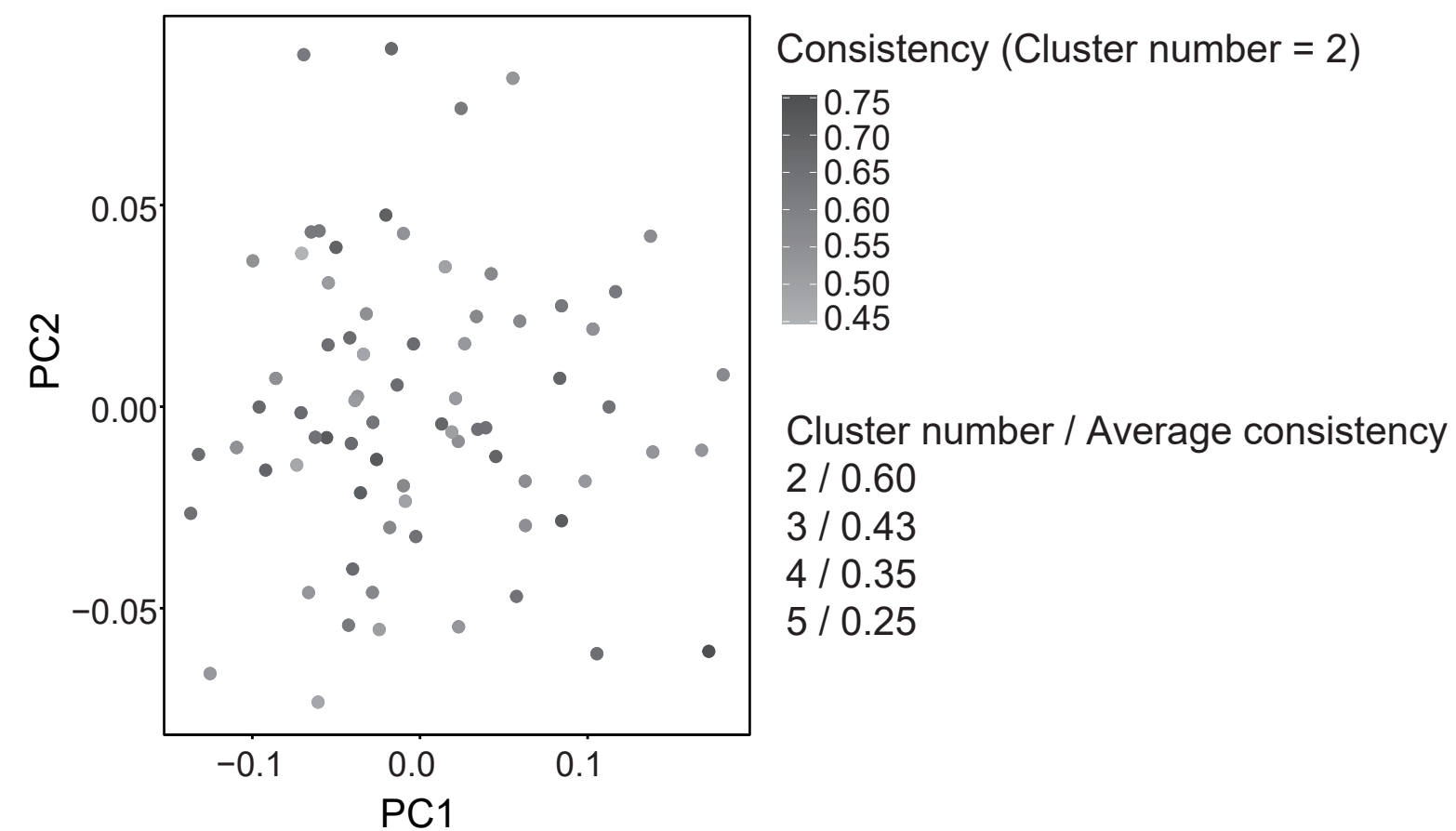

## B Hindgut

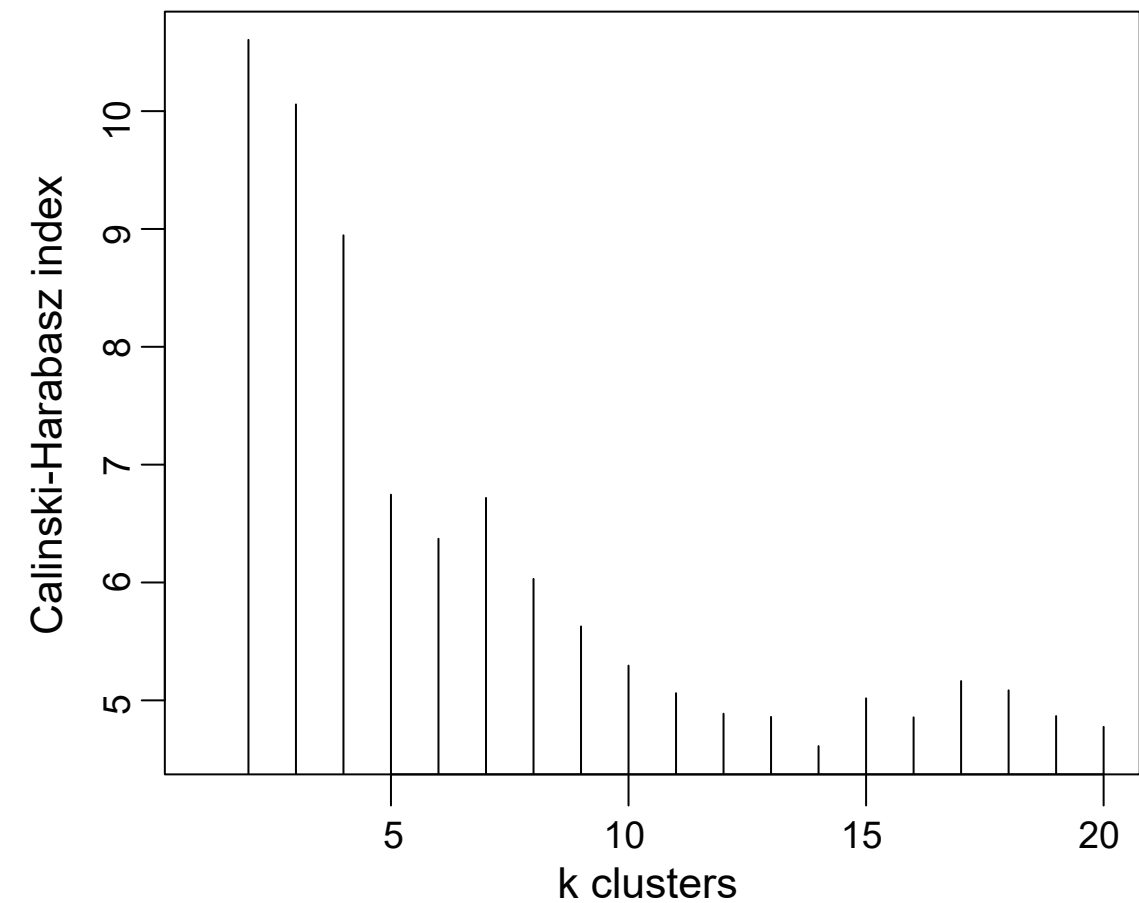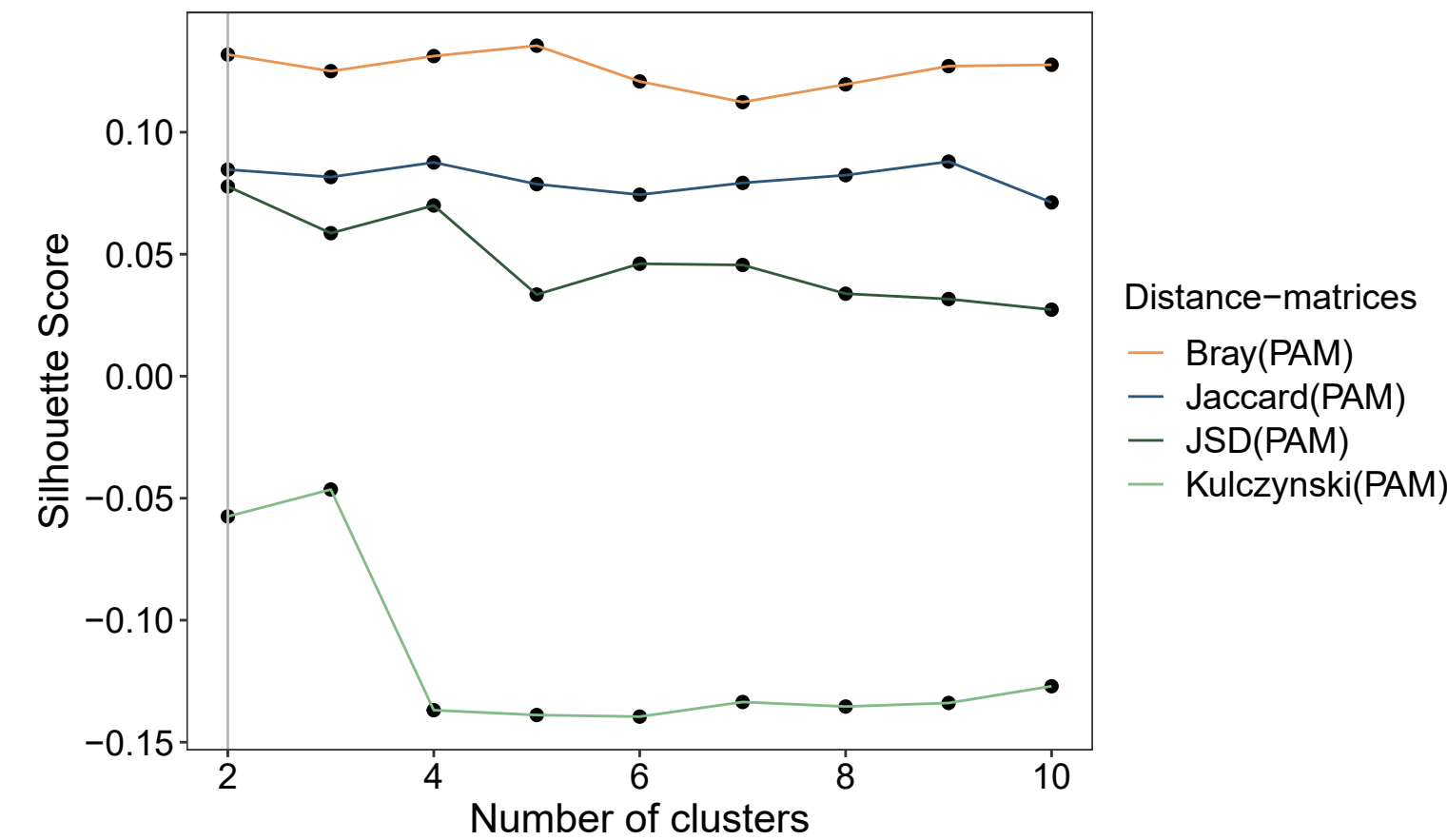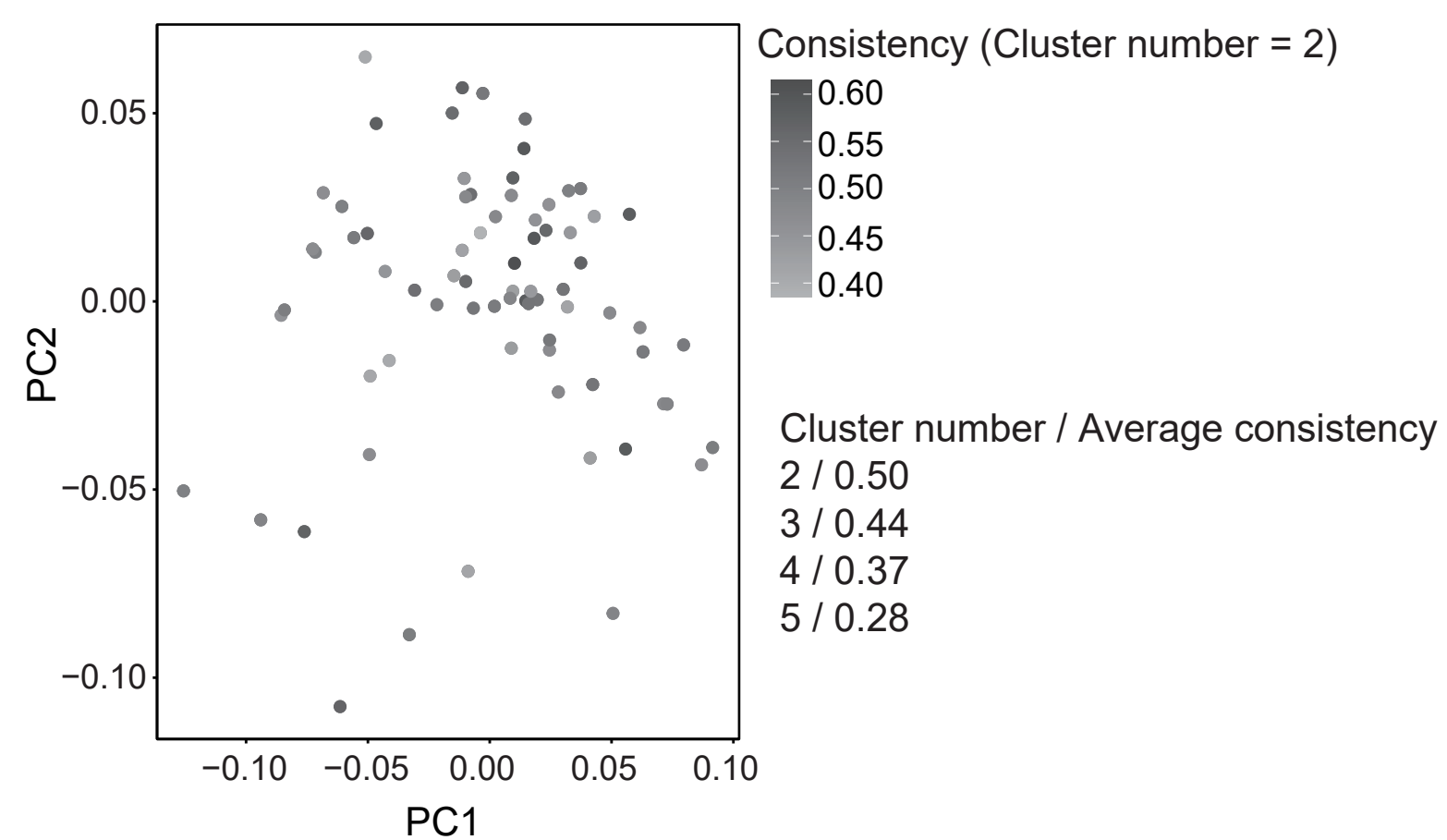

Supplement: Hao_et_al_Fig_S1_ycaf130 [file hao_et_al_fig_s1_ycaf130.pdf]

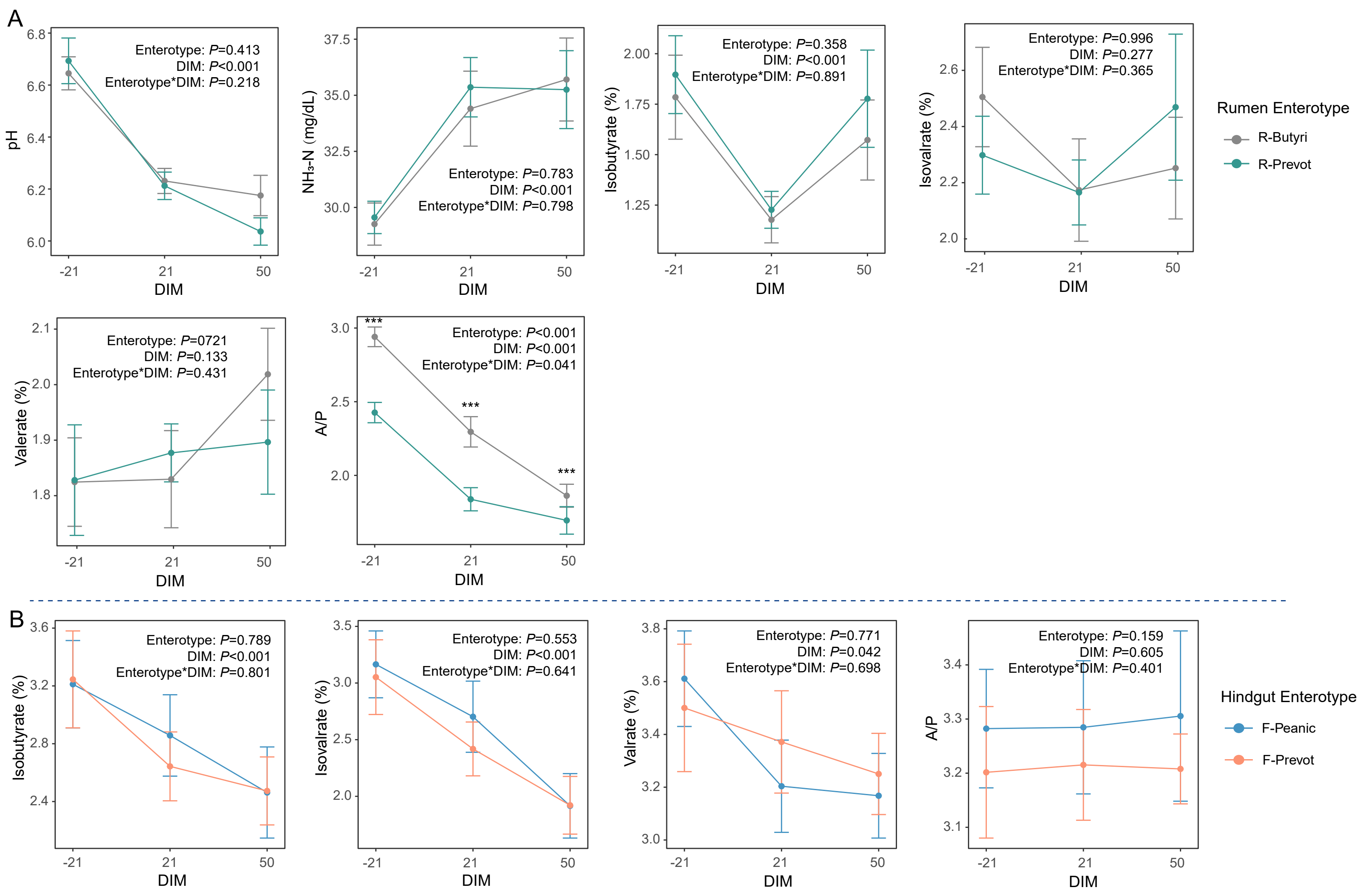

Supplement: Hao_et_al_Fig_S2_ycaf130 [file hao_et_al_fig_s2_ycaf130.pdf]

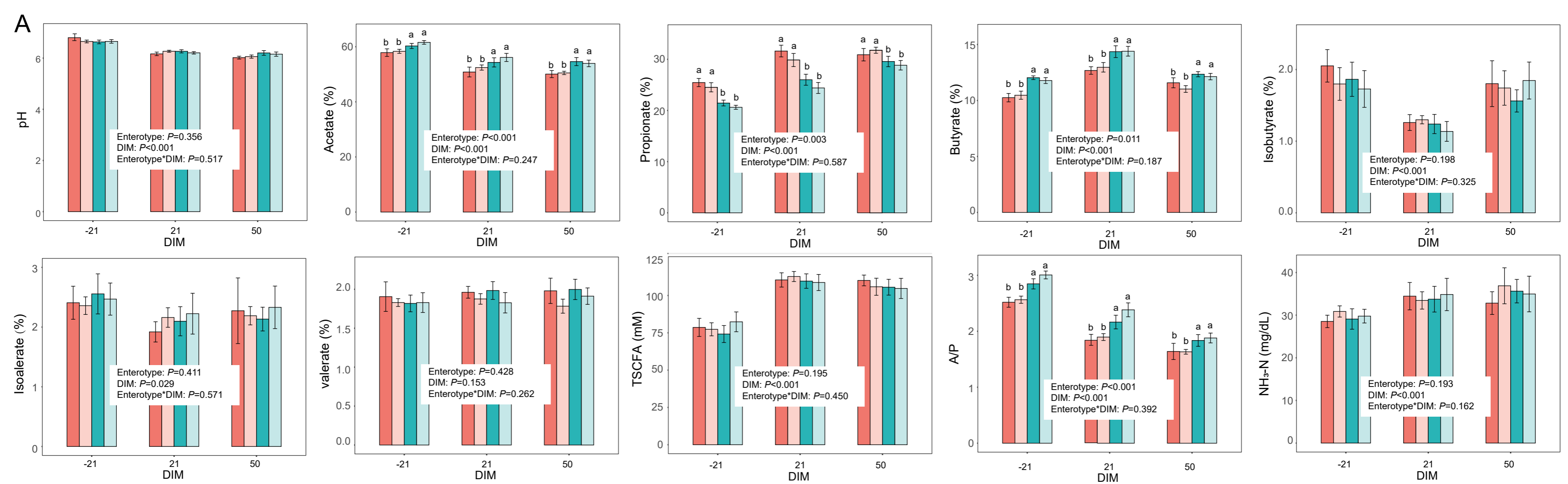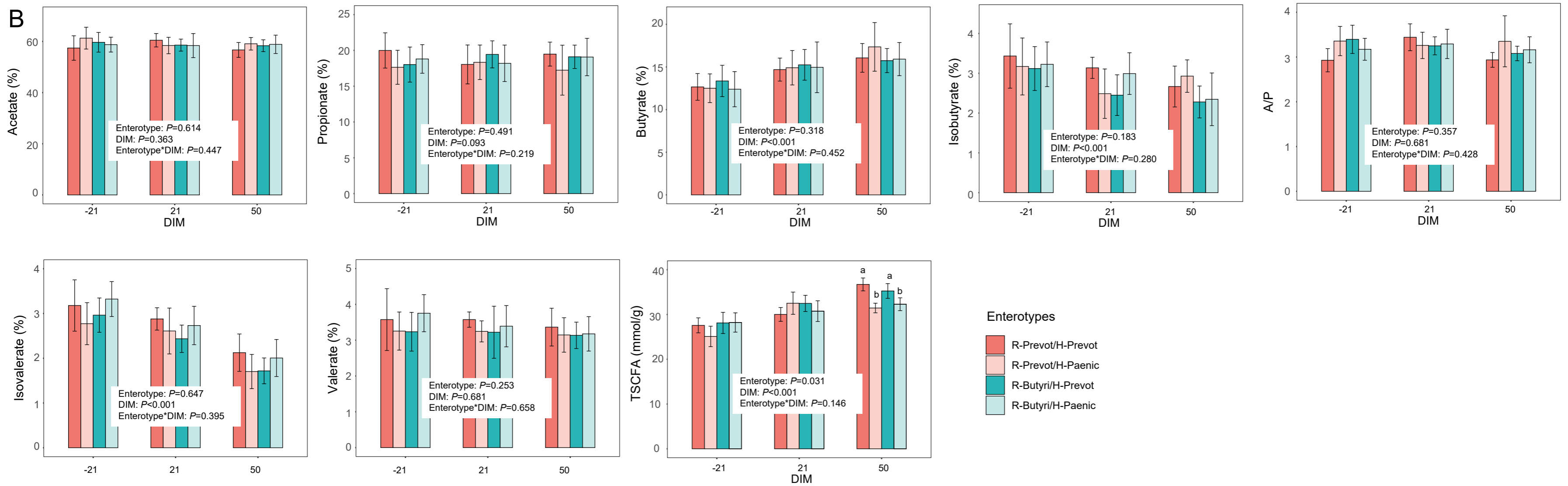

Supplement: Hao_et_al_Fig_S3_ycaf130 [file hao_et_al_fig_s3_ycaf130.pdf]

A

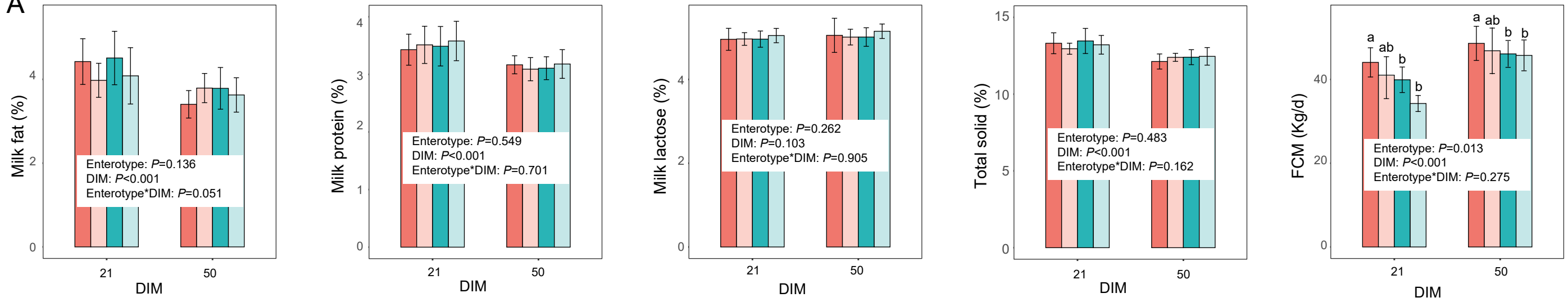

B

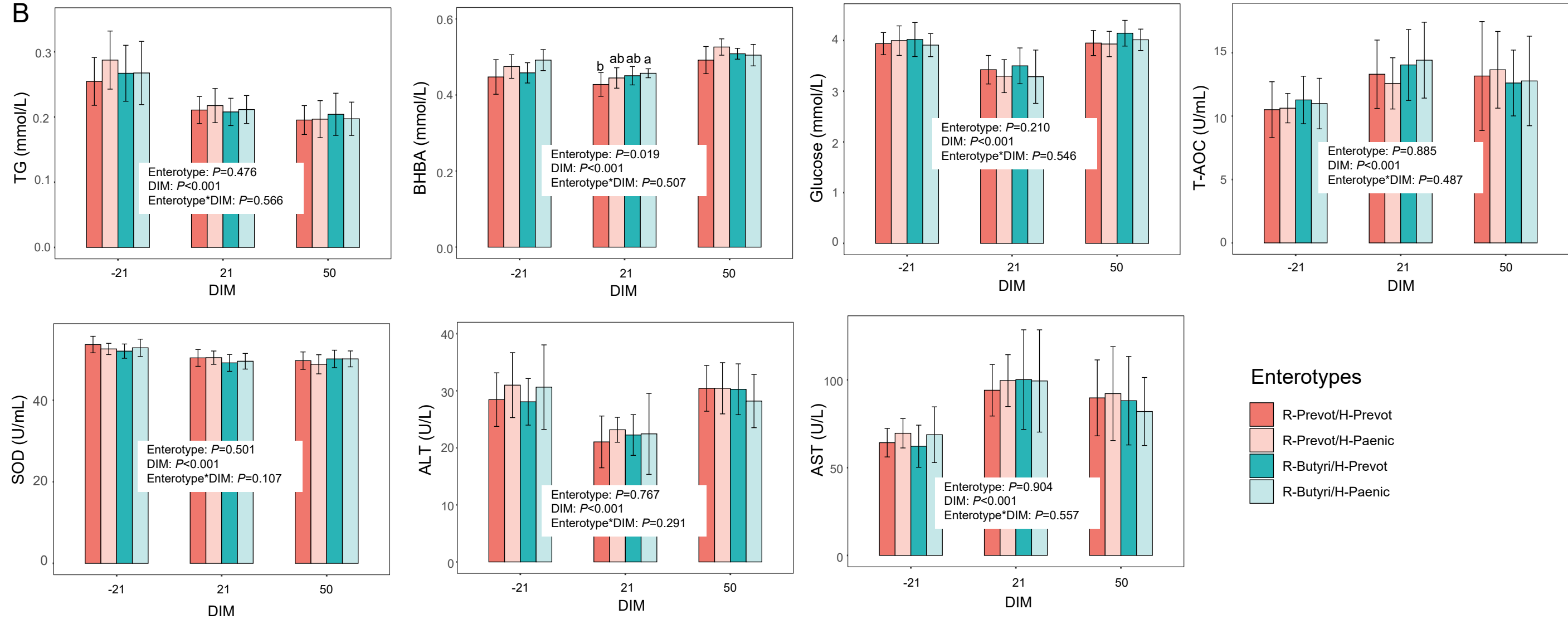

Supplement: Hao_et_al_Fig_S4_ycaf130 [file hao_et_al_fig_s4_ycaf130.pdf]
