# Supplementary material for: Temporal profiling of rumen and hindgut microbiota revealed enterotypes affecting the microbial interactions and assembly in the gut of dairy cows
Source: ISME Commun. 2025 Aug 2;5(1):ycaf130. doi: 10.1093/ismeco/ycaf130 (PMC12376038; doi:10.1093/ismeco/ycaf130)
Supplement: Supplement_Text_S1_ycaf130 [file supplement_text_s1_ycaf130.docx]

**Materials and Methods**

***The procedure for amplicon generation:***

Specifically, PCR amplification was performed with the following conditions: initial denaturation at 95 °C for 3 mins; followed by 27 cycles of denaturation at 95 °C for 30 s, annealing at 55 °C for 30 s, and elongation at 72 ℃ for 45 s, and last a final elongation at 72 ℃ for 10 mins. Negative control with sterile water was included during DNA extraction and subjected to qPCR during amplicon preparation to check for contamination, using the same primers and conditions as for the samples.

**The calculations for the network topology, identification of network specialists and generalists, network robustness, as well as the competitive and cooperative interactions among taxa:**

Specifically, the node number and edge number was counted directly. The average degree was calculated as the average number of edges per node. The edge density was calculated as the proportion of actual edges in a network relative to the total possible edges. It is calculated using the formula:

$$Edge Density=\frac{2E}{N(N-1)}$$

where: *E* = The number of observed edges in the network; *N* = The number of nodes in the network; *N*(*N*−1) = The maximum possible number of edges in an undirected network. A higher number of nodes, edges, average degree, and edge density suggests a greater level of connectivity among taxa, indicating stronger co-occurrence patterns within the community.

Network modularity was calculated using “igraph” within the R package (version: 1.3.5). Based on the within-module connectivity (Zi) and among-module connectivity (Pi), taxa were assigned to specialists and generalists. Specialists included peripheral taxa (Zi < 2.5, Pi < 0.62), which were only connected within a module with at least 60% links [26]; generalists referred to the microbial taxa that were highly connected with others both within and among modules (Network hubs: Zi > 2.5, Pi > 0.62), within a module (module hubs: Zi > 2.5, Pi < 0.62), and among the different modules (connectors: Zi < 2.5, Pi > 0.62) [26]. Additionally, robustness was calculated to test the network stability on the basis of simulation [27, 28] that 50% of network generalists were randomly removed [29] and the proportion of the remaining nodes was reported as the network robustness. To get to know the degree of competitive interactions or cooperative behaviors of ruminal and fecal bacterial communities in different enterotype cows, the negative and positive cohesions in each sample were calculated based on the abundance-weighted, null model-corrected positive and negative correlations, respectively [30]. Negative cohesion, derived from negative pairwise correlations, could reflect the degree of competitive behaviors (such as competition for resources or inhibitory effects) among the community members. Positive cohesion, derived from positive pairwise correlations, could reflect the degree of cooperative behaviors (such as cross-feeding: where one species produces metabolites beneficial to another or mutualistic interactions) among the community members [30]. Specifically, the correlation matrix calculation was based on pearson correlation coefficients. Secondly, a null model correction was applied by shuffling taxon abundances across samples while preserving abundance distributions, generating expected correlations over 200 iterations to account for inherent data structure. After getting the null model-corrected correlations, the taxon connectedness was calculated by the mean of its corrected positive/negative correlations with all other taxa. Finally, the sum of each taxon’s relative abundance was multiplied by its positive/negative connectedness within a sample to get its positive/negative cohesion value.

**The calculation of the assembly process:**

The detailed calculation process of each assembly process was as following: deterministic and stochastic processes were quantified using the beta net relatedness index (βNRI), which measures phylogenetic turnover relative to a null model. When |βNRI| > 1.96, the assembly process was classified as deterministic. Specifically: if βNRI > 1.96, the process was classified as heterogeneous selection; and if βNRI < -1.96, the process was classified as homogeneous selection. When -1.96 < βNRI < 1.96, the Raup-Crick (RC) metric was further calculated to assess taxonomic turnover relative to a null model: if RC < -0.95, the process was classified as homogenizing dispersal; if RC > 0.95, the process was classified as dispersal limitation; and if |RC| ≤ 0.95 and |βNRI| ≤ 1.96, the process was classified as drift and other processes. The significance of the relative importance of ecological processes between different enterotypes was calculated by permutational *t* test (1000 times).
