# Supplementary material for: Temporal profiling of rumen and hindgut microbiota revealed enterotypes affecting the microbial interactions and assembly in the gut of dairy cows
Source: ISME Commun. 2025 Aug 2;5(1):ycaf130. doi: 10.1093/ismeco/ycaf130 (PMC12376038; doi:10.1093/ismeco/ycaf130)
Supplement: Supplement_figures_tables_and_text_information_ycaf130 [file supplement_figures_tables_and_text_information_ycaf130.docx]

**Supplement figures, datasets, and text:**

**Figure S1**: The robustness of rumen and hindgut enterotype enterotypeing. (A) The optimal enterotypeing number was determined by Calinski-Harabasz index. (B) The optimal enterotypeing number was further verified by silhouette score in different method-based distance-matrix, which included Bray-Curtis, Jaccard, Kulcxynski, and Jensen-Shannon distance (JSD). (C) The optimal enterotypeing number was determined by bootstrapping stability analysis.

**Figure S2:** The difference in fermentation parameters between the two enterotypes in rumen (A) and hindgut (B), respectively. The rumen and hindgut fermentation parameters were analyzed using mixed model. Fixed effects included the different combination of enterotypes, DIM, and their interactions. Cows were included as a random effect. In addition, a preplanned t-test was used to evaluate differences in rumen and hindgut fermentation parameters between different enterotypes at each lactation stage. DIM: days in milk. NH_3_-N: ammonia nitrogen. A/P: acetate/propionate ratio. *: *P*<0.05, **: *P*<0.01, and ***: *P*<0.001.

**Figure S3:** The difference in fermentation parameters in rumen (A) and hindgut (B) among the different combinations of rumen and hindgut enterotypes. The fermentation parameters were analyzed using mixed model. Fixed effects included the different combination of enterotypes, DIM, and their interactions. Cows were included as a random effect. The analysis of Variance (ANOVA) test was employed to assess variations in these parameters among the cows with different combinations of rumen and hindgut enterotypes at each lactation stage. The different letters indicates a significant difference among these groups (*P* < 0.05). DIM: days in milk. NH_3_-N: ammonia nitrogen. A/P: acetate/propionate ratio. TSCFA: total short chain fatty acid.

**Figure S4:** The difference in fermentation parameters in milk production (A) and serum parameters (B) among the different combinations of rumen and hindgut enterotypes. The measurements were analyzed using mixed model. Fixed effects included the different combinations of enterotypes, DIM, and their interactions. Cows were included as a random effect. The analysis of Variance (ANOVA) test was employed to assess variations in these parameters among the cows with different combinations of rumen and hindgut enterotypes at each lactation stage. The different letters indicates a significant difference among these groups (*P* < 0.05). FCM: fat corrected milk. TG: triglyceride. BHBA: β-hydroxybutyrate. T-AOC: total antioxidant capacity. SOD: superoxide dismutase. ALT: alanine transferase. AST: aspartate transaminase.

**Data Set S1:** The ingredients and chemical composition of diet for the experiment dairy cows.

**Data Set S2:** The microbial α diversity, network’s robustness, and cohesion, as well as the fermentation parameters in rumen and hindgut between different enterotypes.

**Data Set S3:** The effects of enterotype, days in milk, and their interactions on the relative abundance of microbial taxa in rumen and hindgut, respectively. Only the bacterial genera that presented in at least 50% samples were shown in the data set S3.

**Data Set S4:** The rumen and hindgut microbiota networks topology and the identified generalists.

**Data Set S5:** The milk production and serum parameters between the different rumen and hindgut enterotypes, respectively.

**Text S1:** The supplement information for the method and materials.
